# Integrative analysis and prediction of human R-loop binding proteins

**DOI:** 10.1093/g3journal/jkac142

**Published:** 2022-06-06

**Authors:** Arun Kumar, Louis-Alexandre Fournier, Peter C Stirling

**Affiliations:** Terry Fox Laboratory, BC Cancer, Vancouver, BC V5Z1L3, Canada; Department of Medical Genetics, University of British Columbia, Vancouver, BC V6T 1Z4, Canada; Terry Fox Laboratory, BC Cancer, Vancouver, BC V5Z1L3, Canada; Interdisciplinary Oncology Program, University of British Columbia, Vancouver, BC V6T 1Z4, Canada; Terry Fox Laboratory, BC Cancer, Vancouver, BC V5Z1L3, Canada; Department of Medical Genetics, University of British Columbia, Vancouver, BC V6T 1Z4, Canada; Interdisciplinary Oncology Program, University of British Columbia, Vancouver, BC V6T 1Z4, Canada

**Keywords:** R-loops, R-loop binding proteins, random forest

## Abstract

In the past decade, there has been a growing appreciation for R-loop structures as important regulators of the epigenome, telomere maintenance, DNA repair, and replication. Given these numerous functions, dozens, or potentially hundreds, of proteins could serve as direct or indirect regulators of R-loop writing, reading, and erasing. In order to understand common properties shared amongst potential R-loop binding proteins, we mined published proteomic studies and distilled 10 features that were enriched in R-loop binding proteins compared with the rest of the proteome. Applying an easy-ensemble machine learning approach, we used these R-loop binding protein-specific features along with their amino acid composition to create random forest classifiers that predict the likelihood of a protein to bind to R-loops. Known R-loop regulating pathways such as splicing, DNA damage repair and chromatin remodeling are highly enriched in our datasets, and we validate 2 new R-loop binding proteins LIG1 and FXR1 in human cells. Together these datasets provide a reference to pursue analyses of novel R-loop regulatory proteins.

## Introduction

R-loops are nucleic acid structures consisting of an RNA:DNA hybrid in the genome and an exposed ssDNA loop on the nontemplate strand. R-loops are thought to primarily form cotranscriptionally, although some compelling instances of R-loop formation in *trans* have been observed (Wahba *et al.* 2013; Feretzaki *et al.* 2020). Early studies linked R-loop functions to the regulation of class-switch recombination in developing B cells (Yu *et al.* 2003). However, research in the past decade has greatly expanded our understanding of the many functions of R-loops. We now know that R-loops are key regulatory intermediates of epigenetic states, telomere maintenance, DNA repair reactions, mitochondrial DNA replication, and more (Aguilera and García-Muse 2012;Crossley *et al.* 2019). At the same time dysregulation of normal R-loop formation and dynamics is associated with genome instability, potentially impacting cancers and diseases of trinucleotide repeat-expansion (Stirling and Hieter 2017; Zhang *et al.* 2020). Given the breadth of these functions and the pervasive transcription of the human genome, there is likely to be a large number of factors that regulate R-loop formation, bind R-loops, and resolve R-loops. To borrow a concept from epigenetics, the network of possible R-loop “writers,” “readers,” and “erasers” is potentially very large.

To shed light on this network, significant efforts have gone into cataloguing R-loop binding proteins (RLBPs). Physical interactions with R-loops have been assessed by immunoprecipitation with the S9.6 monoclonal antibody in the presence or absence of synthetic RNA:DNA competitors followed by mass spectrometry (Cristini *et al.* 2018). This study in HeLa cells identified hundreds of candidate R-loop interacting proteins including many known interactors, and focused on validation of DHX9 as a novel R-loop reader in transcription termination, and eraser in genome stability maintenance (Cristini *et al.* 2018). A similar approach was followed in mouse embryonic stem cells to reveal nonredundant roles of DEAD-box proteins in R-loop modulation (Wu *et al.* 2021), and a related study used synthetic RNA:DNA hybrids conjugated to beads to isolate hybrid binding proteins from B-cell lysates (Wang *et al.* 2018). Overall, these 3 studies that rely on immunoprecipitation coupled with mass spectrometry (IP-MS) identified hundreds of candidate’s RLBPs and overlapped by >200 proteins creating a higher confidence set of proteins that may preferentially recognize R-loops. In parallel, 2 studies applied a proximity-labeling approach to characterize the R-loop interactome where biotinylated proteins were purified using streptavidin affinity coupled with mass spectrometry (Prox-MS) (Mosler *et al.* 2021; Yan *et al.* 2022). These Prox-MS studies revealed a new overlapping set of R-loop interactors (>100 proteins) indicating that while IP-MS techniques have their advantages, they enrich for stable R-loop-protein interactions and might lose transient interactions which Prox-MS can capture.

Although the IP-MS and Prox-MS studies have expanded our understanding of the breadth of the R-loop interactome, intersecting all current RLBP proteomic screens results in a surprisingly small overlap (12/1,623; Supplementary Table 1). This could be partially explained by differences in cell lines, purification schemes, and mass spectrometry approaches, but also highlights the need for an integrated approach to accurately predict RLBPs. While each screen reveals novel and interesting new interactors of R-loops, the R-loop binding likelihood of any given protein in the human proteome is currently unknown. Here, building on a set of high-confidence RLBPs, we extract common protein features at the amino acid sequence level, and at higher levels such as protein domain organization or function (Lv *et al.* 2019; Kuechler *et al.* 2020; van Mierlo *et al.* 2021). Based on this analysis, we use random forest (RF) machine learning classifiers to score R-loop binding potential for the human proteome using both IP-MS and Prox-MS as different training datasets. Coupling our 2 machine learning algorithms, we present a partly overlapping resource of candidate RLBPs. While it is unlikely that all of these proteins directly influence R-loops, this dataset provides a resource for ranking candidate genes implicated in R-loop regulated processes such as telomere maintenance, RNA processing, transcription termination, DNA repair, DNA replication, and the regulation of epigenetic states. Our analyses point to expanded representation for known R-loop regulatory complexes such as the spliceosome or DNA repair machinery. We also validate the association and regulatory effect on R-loops for 2 proteins, LIG1 and FXR1, not previously associated with R-loops. Together these analyses provide a foundation for new understanding of the varied functions of RNA:DNA hybrids in the human genome.

## Materials and methods

### Reference databases

Datasets for curating the IP-MS RLBPs list were taken from Cristini *et al.* (2018), the R-loop attracted proteins dataset from Wang *et al.* (2018) and Wu *et al.* (2021) while the Prox-MS RLBPs list were taken from Mosler *et al.* (2021) and Yan *et al.* (2022). Protein domain information was extracted from Pfam version 32.0 using an *E* value <0.001 (El-Gebali *et al.* 2019). Proteins longer than 5,000 amino acids were excluded from this analysis. Protein abundance was mined from PaxDB 4.0 protein abundance whole-organism integrated database (Wang *et al.* 2015). All values from PaxDB are in parts per million. The nucleic-acid binding list was curated and compiled using DNA-binding and RNA-binding lists from Uniprot (UniProt Consortium 2019) and Interpro (Blum *et al.* 2021) supplemented with proteins identified in Beckmann *et al.* (2015) as RNA-associated to make a total of 3,622 proteins. Annotated phosphorylation site data for each protein was mined from the dbPTM database (Huang *et al.* 2019). Protein association networks for the RLBP were created using the current STRING database version 11.0 (Szklarczyk *et al.* 2019). GO pathway enrichment analysis was performed using DAVID v6.8 with respective controls (Huang *et al.* 2009).

### Protein features and data visualization

Protein lengths and percentage of charged amino acids were calculated using in-house R scripts. The R package “Peptides” was used to calculate aliphatic index and GRAVY scores were calculated by using the Kyte-Doolittle algorithm (Kyte and Doolittle 1982). Solubility scores were calculated using CamSol with default settings of pH = 7.0 and proteins with nonstandard amino acids were rejected (Sormanni *et al.* 2015). The overall solubility score for each sequence was used for further analysis. Disorder percentage was calculated using DISOPRED3 (Jones and Cozzetto 2015). Low-complexity region (LCR) percentage was calculated using the fLPS software by predicting compositional biases for the whole protein sequence and dividing it by the total length of each protein (Harrison 2017). Pi–pi interaction tendency was evaluated using PScore (Vernon *et al.* 2018). Any additional analysis and data visualization was performed using custom R scripts (Version 3.6.3).

### RF classifier for R-loop binding prediction

An RF classifier was generated using the “Caret” open-source package in R (https://cran.r-project.org/web/packages/caret/index.html) and was fed the following features for each protein—Length, Aliphatic index, GRAVY, Abundance, CamSol, Disorder and LCR percentage, PScore, capability to bind to nucleic acids, Phosphosite data, and the amino acid makeup for each of the 20 standard amino acids. Feature selection was performed using the Variable Importance evaluation function in Caret and the wrapper algorithm Boruta (Kursa 2014). Both analyses revealed high dependency on abundance and nucleic acid binding capabilities of RLBPs. Using only these features, however, could result in a large number of false positives as the model would not be able to differentiate between other possible features of the protein. Feature selection was therefore not used.

For the training and testing datasets, only proteins that had values for all 30 features were chosen. Proteins present in the IP-MS and Prox-MS RLBP lists were used as the positive class while the rest of the whole proteome was chosen as the negative class. In order to avoid a class imbalance and maintain a 1:1 ratio between the positive and negative class, the remaining whole proteome was randomly shuffled to choose 300 proteins at a time for IP-MS, and 100 proteins for Prox-MS and 100 such shuffles were performed to avoid bias. This generated 200 different RF models for each proteomic approach (100 for IP-MS, 100 for Prox-MS), each with its own training and testing dataset. The dataset was then split into 80% training and 20% testing and the RF algorithm was tuned using a 10-fold cross-validation approach to avoid overfitting. Additionally, the total number of features at each node (mtry) was made dependent on the highest area under the curve (ROC-AUC) to maximize ROC-AUC, thus improving prediction power. Each protein was assigned a probability score between 0 and 1 such that proteins with probabilities greater than or equal to 0.5 were classified as RLBPs. Applying an easy ensemble approach, the probabilities from all 100 models per approach were averaged for each protein and subjected to thresholds. Proteins with an average probability >0.8 over 100 models were extracted for higher confidence resulting in a final set of 665 proteins for the IP-MS trained algorithm, and 488 for Prox-MS trained algorithm with an overlap of 288 proteins between the two. Scripts used for RF tuning, development and prediction can be found on Github (https://github.com/arunk95/RLBP_Prediction).

### RLBP network analysis

Functional gene interaction network analysis was performed and visualized using Cytoscape [v3.8.2 (Shannon *et al.* 2003**)** and GeneMANIA (v3.5.2) (Montojo *et al.* 2010)]. Genes were manually annotated based on the literature and corresponding GO terms (biological processes and molecular functions) using g: Profiler (*P* < 0.05, with Benjamini–Hochberg FDR correction) (Raudvere *et al.* 2019).

### Cell culture, siRNA transfection, and drug treatments

HeLa cells were cultivated in Dulbecco’s modified Eagle’s medium (Stemcell technologies) supplemented with 10% fetal bovine serum (Life Technologies) in 5% CO_2_ at 37°C. For RNA interference, cells were transfected with siGENOME-SMARTpool siRNAs from Dharmacon (Nontargeting siRNA Pool #1 as si-Cont, ON-TARGETplus Human LIG1 3978 siRNA SMARTpool, and ON-TARGETplus Human FXR1 8087 siRNA SMARTpool). Transfections were performed with Dharmafect1 transfection reagent (Dharmacon) according to the manufacturer’s protocol and harvested 48 h after siRNA transfection. For drug treatments, cells were treated 25 µM L82-G17 (Aobius, AOB31453) for 4 h prior to fixation.

### Proximity ligation assay

Proximity ligation assay (PLA) experiments were performed using the Duolink PLA kit (Millipore Sigma). For all PLA experiments, cells were grown on coverslips before fixing. For S9.6 staining, cells were fixed with ice-cold methanol for 10 min and permeabilized with ice-cold acetone for 1 minute. After permeabilization, cells were washed with PBS and blocked for 1 h at RT in Duolink Blocking Solution. Cells were then incubated with primary antibodies (S9.6 Millipore MABE1095 + anti-LIG1 Abcam ab177946 or anti-FXR1 Abcam ab155124) diluted in Duolink Antibody Diluent overnight at 4°C. Cells were washed twice (5 min) in µl/cover slip, 1:4 PLA probe in PLA Antibody Diluent. Cells were washed (2× 5 min) with PLA Wash Buffer A, after which they were incubated 30 min at 37°C with PLA ligation mix (15 µl/cover slip, 1:40 PLA ligase 40× in 1:5 ligation buffer 5× in Ultra H_2_O). Cells were washed (2× 2 min) with PLA Wash Buffer A and incubated 100 min at 37°C with PLA Amplification mix (15 µl/slide, 1:80 polymerase solution in 1:5 amplification stock in ultra H_2_O). The cells were washed (2× 10 min) in PLA Wash Buffer B. After an additional wash (1 min) in PLA Wash Buffer B 0.01×, the slides were mounted in Duolink Mounting Media with DAPI. Imaging was performed on a Leica Dmi8 microscope at 100×. ImageJ was used for image processing and quantification (Schneider *et al.* 2012). For in vitro RNaseH, RNaseIII, and RNaseT1 treatment, cells were treated with RNaseH (New England Biolabs, M0297S) for 2 h, or ShortCut RNaseIII (New England Biolabs, M0245S) or RNaseT1 (ThermoFisher Scientific, EN0541) for 30 min at 37°C after permeabilization before blocking.

### Immunofluorescence

For all immunofluorescence experiments, cells were grown on coverslips overnight before transfection with 50 µM siRNA (siCTRL, siLIG1, or siFXR1). Forty-eight hours post-transfection, the cells were fixed with ice-cold methanol for 10 min and permeabilized with ice-cold acetone for 1 min. After permeabilization, cells were washed with PBS and blocked in 3%BSA, 0.1% Tween 20 in 4× saline sodium citrate buffer for 1 h at RT. Cells were then incubated with primary antibody (S9.6, Millipore) overnight at 4°C. Following 2 PBS washes and an additional 20 min blocking step, the cells were then incubated with Alexa-Fluor-568-conjugated secondary antibodies for 1 h at RT. After washing 3 times with PBS, the cells were stained with DAPI before mounting and imaging on a LeicaDMI8 microscope at 100×. ImageJ was used for image processing and quantification of nuclear S9.6 signal (Schneider *et al.* 2012). For in vitro RNaseH, RNaseIII, and RNaseT1 treatment, cells were treated according to the manufacturer’s guidelines with in vitro RNaseH (New England Biolabs, M0297S) for 2 h, or ShortCut RNaseIII (New England Biolabs, M0245S) or RNaseT1 (ThermoFisher Scientific, EN0541) for 30 min at 37°C after permeabilization before blocking.

### Western blotting

Whole-cell lysates were prepared with RIPA buffer containing protease inhibitor (Sigma) and phosphatase inhibitor (Roche Applied Science) cocktail tablets and the protein concentration were determined by Bio-Rad Protein assay (Bio-Rad). Equivalent amounts of protein were resolved by SDS-PAGE and transferred to polyvinylidene fluoride microporous membrane (Millipore), blocked with 1.5% BSA in H20 containing 0.1% Tween-20 (TBS-T), and membranes were probed with the following primary antibodies: anti-LIG1 (Abcam ab177946) (1:1,000), anti-FXR1 (Abcam ab155124) (1:1,000), and anti-GAPDH (Thermo Scientific MA5-15738) (1:3,000). Secondary antibodies were conjugated to horseradish peroxidase (HRP) and peroxidase activity was visualized using Chemiluminescent HRP substrate (Thermo Scientific).

### DRIP and ChIP qPCR

DRIP was performed using the SimpleCHIP enzymatic chromatin IP kit (Cell Signaling Technologies) in accordance with the manufacturer’s instructions with some modifications. HeLa cells were seeded at 1× 10^6^ in 10 cm plates and transfected the following day with siRNAs (siCTRL, siLIG1, siFXR1). Forty-eight hours after transfection, the cells were crosslinked in 1% formaldehyde for 10 min, followed by 2 min incubation in 1× glycine solution (CST#7005). Cells were washed in 10 ml ice-cold 1× PBS twice and scraped into 0.5 ml ice-cold 1× Buffer A [with 500 µM DTT and 1× protease inhibitor cocktail (PIC) (CST#7012) per IP prep in sonication tubes]. Nuclei were pelleted and resuspended in 150 µl of 1× ChIP buffer and 1× PIC, followed by incubation on ice for 10 min. DNA was sonicated on Q Sonica Sonicator Q700 for 8 min (30 s ON, 20 s OFF) at 100 µm amplitude to fragment DNA. Lysates were clarified with centrifugation and the supernatant was collected DRIP and stored at −80°C until further use. DNA samples for DRIP were treated with RNAse A (CST#7013) followed by proteinase K (CST#10012) overnight at 65°C. DNA samples were purified with Cell Signaling spin columns (#14209S). DNA concentration was measured using NanoDrop One/Onec. DNA samples were used for DRIP analysis, where 2% input sample was isolated and DNA was separated into untreated and RNaseH-treated groups. One microliter of 5,000 units/ml RNaseH (NEB, M0297L) was used per micrograms of DNA and incubated for 48 h at 37°C. ChIP-Grade protein G magnetic beads (Cell Signaling, 9006S - 25 μl per IP) were preblocked in preblocking buffer (PBS/EDTA containing 0.5% BSA) for 1 h rotating at 4°C. Beads were immobilized with S9.6 antibody (MABE1095) using 5 µg per IP, in 1× ChIP buffer and 1× PIC for 4 h rotating at 4°C. DNA was then added to the beads/Ab complex and incubated overnight with rotation at 4°C. Samples were washed 3 times with 1 ml low salt buffer (0.1% SDS, 1% Triton X-100, 2 mM EDTA pH 8.0, 20 mM Tris−Cl pH 8.0, and 150 mM NaCl), 1 time with 1 ml high salt buffer (0.1% SDS, % Triton X-100, 2 mM EDTA pH 8.0, 20 mM Tris-Cl pH 8.0 ,and 500 mM NaCl), and eluted in 80 µl of 1× elution buffer (CST#7009) for 30 min at 65°C vortexing intermittently. Beads were pelleted and supernatant was purified with Cell Signaling spin columns (CST#14209S). DRIP-qPCR primer sequences can be found in Supplementary Table 5. qPCR was performed on AB Step One Plus real-time PCR machine (Applied Biosystems) using Fast SYBR Green Master Mix (Applied Biosystems).

## Results

### Defining characteristics of consensus R-loop interacting proteins

**Fig. 1.**
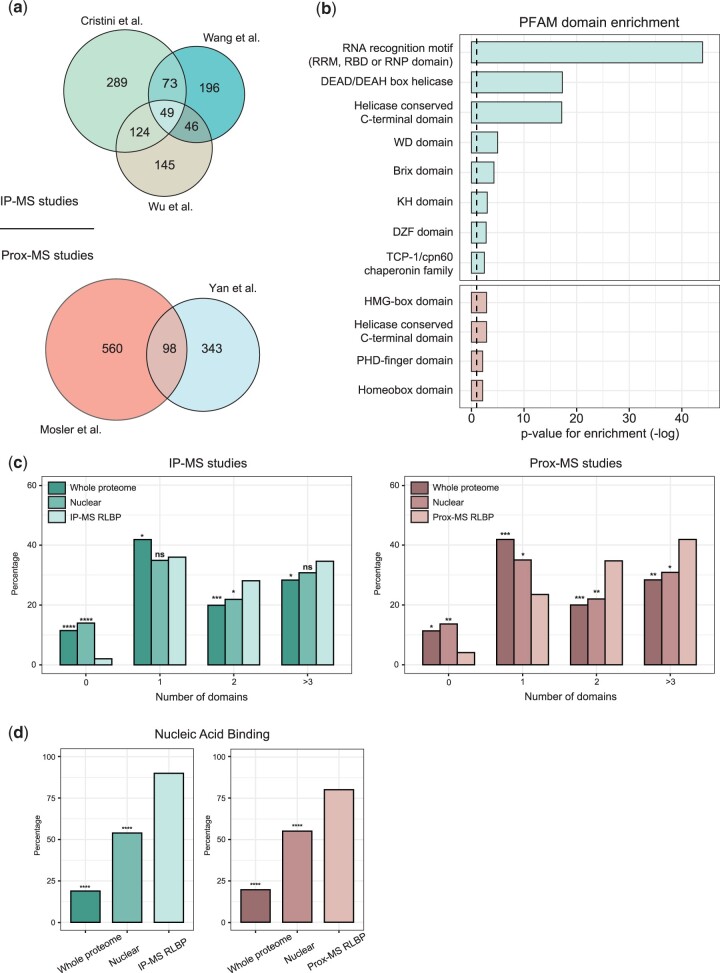
Higher-order features of RLBPs. a) Venn diagram of candidate RLBPs identified in published proteomic studies. b) Enrichment analysis of PFAM domains enriched within the IP-MS and Prox-MS overlaps (False discovery rate adjusted *P*-value <0.01). c) Comparison of domain numbers between the whole proteome, nuclear proteome, and respective RLBP datasets. d) The proportion of proteins in each category encoding a nucleic acid binding domain. For (c) and (d), ****P < 0.0001, **P < 0.01,*P < 0.1, ^ns^*P* > 0.1 Fisher’s exact test.

We set out to understand common features of RLBPs by analyzing their protein sequences and higher-order features. To develop a consensus dataset, we first intersected 3 studies that document R-loop interacting proteins using orthogonal enrichment approaches followed by mass spectrometry (IP-MS and Prox-MS). Upon intersecting the Cristini *et al.* (2018) dataset (469 proteins), Wang *et al.* (2018) dataset (313; subset of proteins attracted to R-loops), and Wu *et al.* (2021) dataset (364 proteins) we found 49 high confidence proteins that preferentially bind R-loops. Additionally, we also chose common proteins between any 2 studies to reveal a highly stringent list of 292 proteins that we classify as RLBPs identified through IP-MS approaches (IP-MS RLBPs) (Fig. 1a; Supplementary Table 1). Similarly, to develop a consensus proximity-based RLBP dataset, we intersected the Mosler *et al.* (2021) dataset (660 proteins) and the Yan *et al.* (2022) dataset (440 proteins) to reveal 101 proteins that have high R-loop binding affinity identified via Prox-MS approaches (Prox-MS RLBPs). Hereon, the terms RLBPs refers to both IP-MS RLBPs and Prox-MS RLBPs unless specified otherwise.

To assess the functional properties of RLBPs, we performed a protein domain enrichment analysis using the Pfam database. As expected, the analysis identified an enrichment for DEAD/DEAH-box domains that have previously been linked to RLBPs (Fig. 1b) (Cristini *et al.* 2018;Wang *et al.* 2018). Interestingly, the IP-MS RLBPs contained multiple RNA-binding domains including the RRM motif and OB-fold domains, while the Prox-MS RLBPs were selectively enriched for HMG-box and Homeobox domains. Additionally, RLBPs are significantly enriched for proteins with 3 or more domains compared with the whole human proteome (Fig. 1c). While RLBPs contain a larger proportion of proteins with 2 domains, the RLBP dataset has relatively few proteins containing one or no domain indicating that RLBPs have a high valency, in that RLBPs can probably form multiple protein–protein interactions. This is further supported by a tightly interconnected protein–protein physical interaction network of RLBPs reported by the STRING database (*P*-value <1.0e^−16^; https://string-db.org/). Since RLBPs must recognize the RNA or DNA region of the R-loop, we hypothesized the RLBPs should be enriched for nucleic acid binding domains. Indeed, when compared with both the whole proteome and the nuclear proteome, there was a strong enrichment for nucleic acid binding domains, supporting that this is an important property of RLBPs (Fig. 1d).

### Protein sequence and functional characteristics of the R-loop binding proteome

**Fig. 2.**
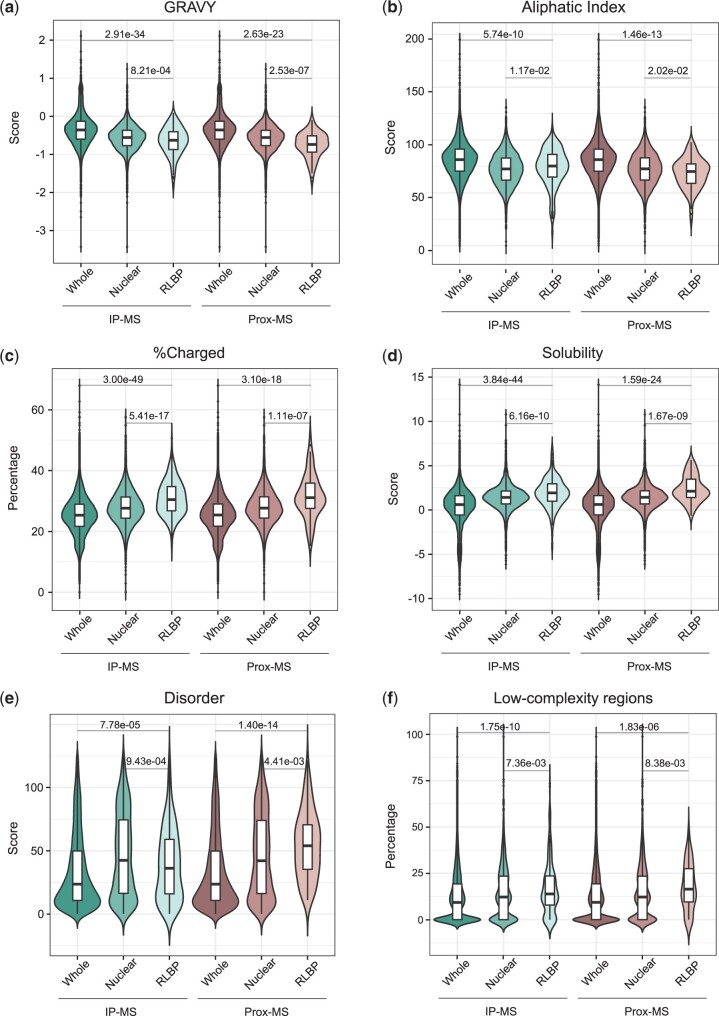
Amino acid sequence characteristics of RLBPs. a) Hydrophobicity and hydrophilicity (GRAVY); b) aliphatic index; c) percentage of charged residues; d) solubility; e) protein disorder; and f) percentage of LCRs were compared for the indicated whole proteome, nuclear proteome, and the respective RLBP datasets. The exact *P*-values resulting from a Mann–Whitney–Wilcoxon test after Bonferroni correction for multiple comparisons are reported.

Next, we investigated the potential for shared protein sequence features amongst RLBPs. First, we assessed the hydrophobic character of each protein by calculating GRAVY (Grand Average of hydropathy) scores (Kyte and Doolittle 1982). On average, the RLBPs have lower GRAVY scores vs the human proteome suggesting a more hydrophilic character that could enable a more flexible protein structure and associate with nucleic acid binding properties (Fig. 2a). We then calculated the aliphatic index, which monitors the volume of A, L, V, and I sidechains, as a proxy for protein thermostability. Consistent with low GRAVY scores, we found that RLBPs have significantly lower indices compared with the rest of the proteome (Fig. 2b). Surprisingly, IP-MS RLBPs have a higher average aliphatic index than the nuclear control, indicating that these proteins might have a characteristic range of value that could be used to differentiate them from other nuclear proteins. On the other hand, Prox-MS RLBPs follow the opposite trend wherein the average aliphatic index is lower than the nuclear proteome. Next, we assessed protein length and found that RLBPs are significantly longer than the average human protein (Supplementary Fig. 1a). Interestingly, the difference between the IP-MS RLBPs and the nuclear proteins was not significant suggesting that their average length might be common to all nuclear proteins and not just the IP-MS RLBPs. We also investigated the amino acid makeup of RLBPs and found that they are enriched for charged amino acids (Fig. 2c). Interestingly, this trend holds true for both positive and negatively charged amino acids (Supplementary Fig. 1, b and c). Overall, we find that a typical RLBP is longer, more stable, more hydrophilic and enriched for charged amino acids when compared with the rest of the human proteome, which might prime them for interactions with other proteins.

We next sought to characterize the biochemical properties of the RLBP dataset. We first measured protein solubility using the CamSol prediction method that utilizes protein sequences to assign solubility scores (Sormanni *et al.* 2015). We found that RLBPs have significantly higher scores, indicating that they are more soluble and may have a lower tendency to form aggregates (Fig. 2d). We then compared protein abundance using the PaxDB database (Wang *et al.* 2015) and found that RLBPs are more abundant than the average of both the whole proteome and nuclear proteins (Supplementary Fig. 1d). Based on this data, RLBPs appear to be abundant, soluble and charged proteins. We wondered if this would affect their propensity to phase-separate. Intrinsically disordered regions (IDRs) and LCRs in proteins have been implicated as key drivers of liquid–liquid phase separation (Alberti *et al.* 2019). These regions do not form canonical 3D structures, making them more flexible and prone to interactions. Previous studies have noted the presence of IDRs in some RLBPs (Wang *et al.* 2018; Dettori *et al.* 2021), and accordingly DISOPRED3 disorder scores show that RLBPs are more disordered than the human proteome (Fig. 2e) (Jones and Cozzetto 2015). However, similar to the aliphatic index (Fig. 2b), we saw a contrast between IP-MS RLBPs and Prox-MS RLBPs when compared with their nuclear proteomic controls. IP-MS RLBPs were more disordered than the whole proteome but significantly lower than their nuclear proteome, while Prox-MS RLBPs were significantly more disordered than both the whole proteome and the nuclear proteome. This clear distinction supports the idea that different proteomic approaches enrich different proteins, and perhaps enrich for different properties. We also measured the presence of LCRs using fLPS, which annotates compositional biases in a given sequence to provide a total number of LCRs in a protein (Harrison 2017). As with protein disorder, RLBPs present a significant enrichment of LCRs compared with the human proteome (Fig. 2f). The trend held true when we measured the propensity to phase separate through π–π interactions by measuring PScore (Supplementary Fig. 1e) (Vernon *et al.* 2018). Finally, we used phosphorylation as a proxy to determine the proportion of the proteome that could be post translationally modified, and observed a significant increase for both IP-MS and Prox-MS RLBPs (Supplementary Fig. 1f) (Huang *et al.* 2019). Overall, we find that the RLBP dataset is more abundant, soluble, disordered, has a higher valency and a greater tendency to be post translationally modified compared with the rest of the proteome.

### An RF classifier of R-loop binding properties in the human proteome

Our data so far suggest that high confidence RLBPs have common sequence features, functional properties, and domain architectures. Thus, we hypothesized that these features could be used to predict new candidate RLBPs in the rest of the human proteome and to increase confidence in selected RLBP candidates detected in only a single study. Using the intrinsic features of RLBPs that we identified, we set out to develop a tool that can assign a score of the likelihood that any given protein in the human proteome is an RLBP. We employed a machine learning approach using the RF algorithm to develop a binary classifier that could yield predictions of the highest confidence. The RF algorithm is a common machine learning method owing to its versatility, and its ability to leverage big data with multiple variables (Chen and Ishwaran 2012). In total, our RF model assesses 30 features including: length, GRAVY, aliphatic index, abundance, CamSol scores, disorder%, LCR%, PScore, ability to bind nucleic acids, Phosphosite%, and the amino acid makeup of all 20 standard amino acids (Supplementary Table 4). We chose not to eliminate any features after performing feature selection to reduce possible false positives (see *Materials and* *methods*).

**Fig. 3.**
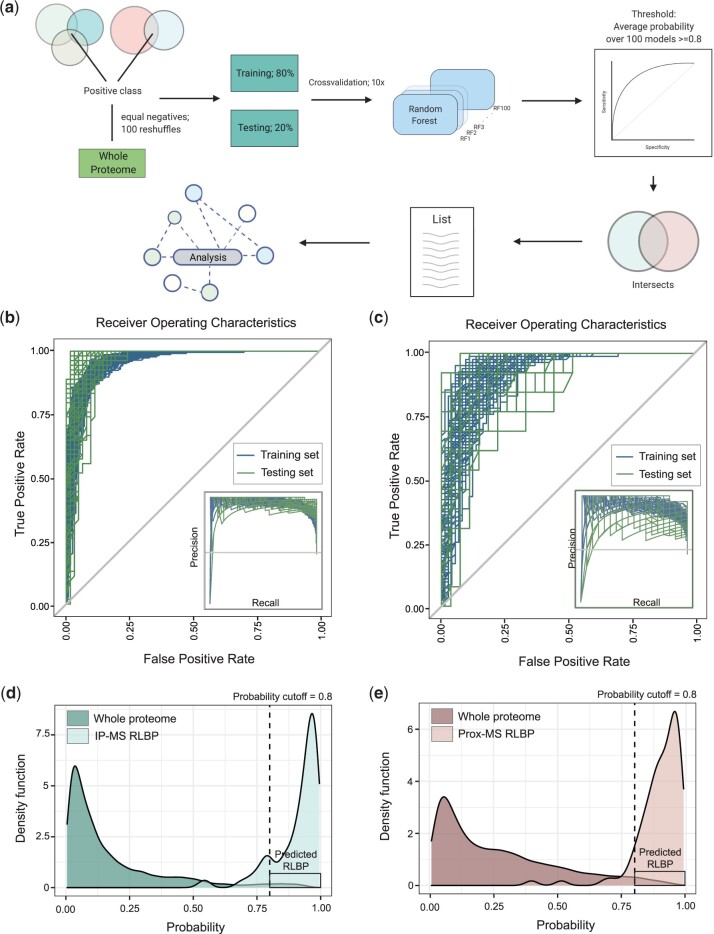
An RF classifier to determine R-loop binding property likelihood. a) Schematic of the machine learning model pipeline (created with BioRender). b, c) Receiving operating characteristics of the training (blue) and testing (green) sets with included precision recall-gain curves for IP-MS RF and Prox-MS RF models respectively. d, e) Whole proteome prediction of RLBP character by both models. The plot shows the probability distribution of the whole proteome and our IP-MS or Prox-MS RLBP training set. The newly predicted RLBPs extracted from the whole proteome are highlighted in the box on the right.

**Fig. 4.**
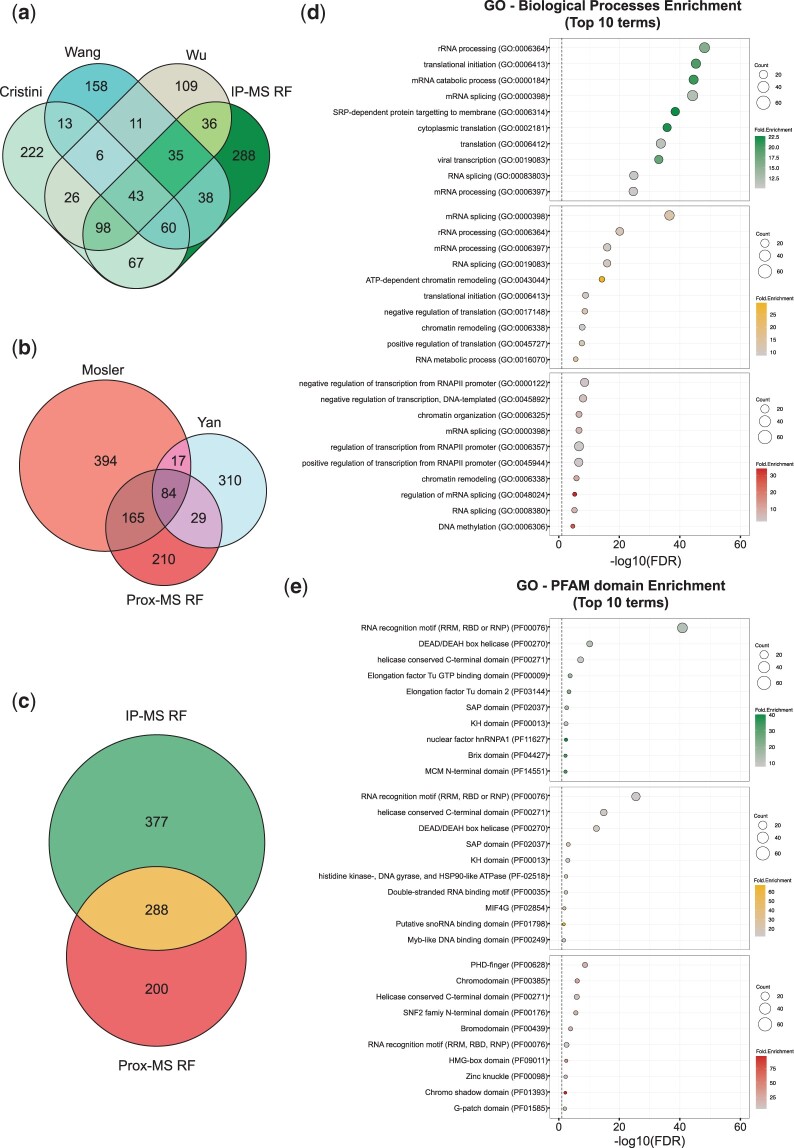
Common characteristics of predicted RLBPs. Venn diagrams showing the overlap between our Machine Learning algorithms and published IP-MS (a) and Prox-MS (b) datasets. c) Venn diagram showing the overlapping proteins predicted by our IP-MS and Prox-MS RF algorithms. Top 10 enriched GO terms, biological processes (d) and PFAM domains (e), identified using David v 6.8 (*P* < 0.05, with FDR correction) among IP-MS RF hits (*n* = 377, green), overlapping hits (*n* = 288, yellow), and Prox-MS RF hits (*n* = 200, red).

Since the IP-MS and Prox-MS methods identified different sets of RLBPs, each with their own characteristic feature ranges, we decided to build two separate RF models: one for each proteomic approach. To train and test our classifier, we chose respective IP-MS and Prox-MS RLBPs as our positive class while the remainder of the whole human proteome made up the negative class. To avoid class imbalance, we employed an easy ensemble approach where we randomly shuffled the negative class 100 times and chose 300 proteins for IP-MS and 100 proteins for Prox-MS (see *Materials and methods*) from each iteration, resulting in 100 different training and testing datasets and therefore 100 different RF models per proteomic approach. Using an 80% training, 20% testing split, along with 10-fold cross-validation, we assessed the performance of our classifiers (Fig. 3a). On average, our IP-MS classifiers achieved an accuracy of 93.3%, an F1 score of 0.931 and a Matthews correlation coefficient (MCC) of 0.869 on the test set whereas the Prox-MS classifiers achieved an accuracy of 82%, an F1 score of 0.805, and MCC of 0.643 (Fig. 3, b and c; Supplementary Table 3). While both models had good performance metrics that supported further analyses, the IP-MS model performed better than the Prox-MS model. This was also evident when comparing the receiver operating characteristics between the 2 models (Fig. 3, b and c). One possible explanation for the difference in performance can perhaps be explained by the difference in sample sizes for the training datasets. Each protein was assigned a probability score between 0 and 1, where proteins ≥0.5 are classified as candidate RLBPs. To determine a probability cutoff for new candidate RLBPs, we plotted the density function for both the RLBPs and the remaining proteome (Fig. 3, d and e). The RLBPs peak at 0.95 whereas the remaining proteome peaks at 0.05. This was not surprising as the RF models should predict RLBPs as positive (i.e. ∼1) and the rest of the proteome as negative (i.e. ∼0). Importantly, the tail end of the whole proteome set falls in a higher probability range indicating newly predicted RLBPs (rectangular box in Fig. 3, d and e). To ensure a stringent cutoff, we chose 0.8 as a conservative threshold to call new candidate RLBPs. Using 0.8 as our cutoff, we present a set of 665 proteins that are predicted as R-loop binding by the IP-MS model and 488 proteins by the Prox-MS model (Fig. 4, a and b). When intersected with the published datasets used to produce the original training lists, the IP-MS model predicts 236 of the 292 proteins used to train the model. Of the remaining 429 proteins, the model successfully predicts 67 proteins found only in the Cristini dataset, 38 found only in the Wang dataset, and 36 proteins found only in the Wu dataset (Cristini *et al.* 2018; Wang *et al.* 2018; ) (Fig. 4a). This represents a significant enrichment (*P* = 3.70e^−64^ Cristini; *P* = 1.20e^−31^ Wang; *P* = 1.90e^−31^ Wu, Fisher’s exact test), suggesting that there are indeed common properties of RLBPs that can be extracted from the proteomic datasets. Consistently, the Prox-MS model predicts 84 of the 101 proteins in the training dataset, 165 proteins only in the Mosler dataset and 29 proteins from the Yan dataset (*P* = 2.30e^−154^ Mosler; *P* = 1.30e^−10^ Yan, Fisher’s exact test) (Fig. 4b). Together, these 335 proteins represent a second set of candidate RLBPs that are both predicted by our feature model, and found in one published proteomic study (Fig. 4, a and b). Additionally, we intersected both IP-MS and Prox-MS RF models to present a final dataset of 288 potential RLBPs (Fig. 4c).

Gene Ontology analysis for biological process enrichment on all 3 datasets (i.e. IP-MS RF, Prox-MS RF, and their overlap) revealed strong enrichment for proteins involved in regulation of mRNA splicing, RNA processing, DNA replication, and various forms of DNA repair (Fig. 4d; Supplementary Table 4). Most of these processes have been implicated in R-loop biology by previous studies (Aguilera and García-Muse 2012; Chang and Stirling 2017; Crossley *et al.* 2019). Unsurprisingly, although there was significant overlap between the 3 analyses, the IP-MS RF and Prox-MS RF were also enriched for different processes. For instance, the Prox-MS RF algorithm identified chromatin remodeling, which reflects the ability of proximity-labeling to possibly enrich for more transient interactors of R-loops and/or identify R-loop regulators outside of the immediate vicinity of R-loops. In accordance, gene ontology analysis for domain enrichment among the 3 datasets shows conserved RNA binding capabilities through RRM domains, while differences between the IP-MS RF and Prox-MS RF datasets pertain primarily to chromatin binding (Fig. 4e;Supplementary Table 4). Similar observations were also reported by Mosler *et al.* (2021) when they compared their proximity-labeling dataset with previous IP-MS studies. To determine whether nucleic acid binding overly skews the RF algorithm, we compared the assessed features between the predicted RLBPs and the remaining nucleic acid binding proteins. Except for length and LCRs for IP-MS and aliphatic index for Prox-MS, there is a significant enrichment for all remaining features supporting our hypothesis that the RLBP dataset is a unique subset of cellular proteins with potential R-loop binding ability (Supplementary Fig. 2, a–i). Together, our model recovers the vast majority of high confidence RLBPs, selects new candidates found in previous mass spectrometry studies, and assigns an RLBP probability to the rest of the human proteome.

### The functional spectrum of candidate RLBPs

**Fig. 5.**
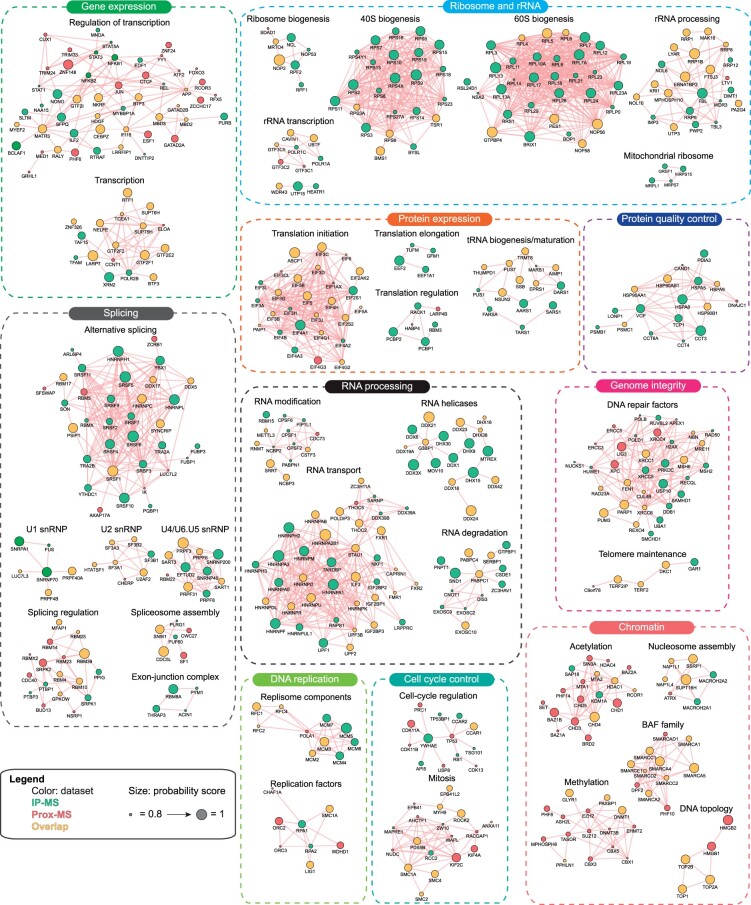
Integrative analysis of enriched complexes involved in R-loop binding. a) Functional interaction network of the combined IP-MS RF and Prox-MS RF predicted RLBPs. Genes were manually annotated based on the literature and corresponding GO terms (biological processes and molecular functions). Edges between nodes indicate physical interactions between proteins (Cytoscape, Genemania).

With both machine learning screens in hand, we undertook a functional network analysis to identify the cellular complexes enriched by the datasets (Fig. 5). To do so, genes were manually annotated based on the literature and corresponding GO terms (biological processes and molecular functions) using g: Profiler (Raudvere *et al.* 2019). This analysis revealed spliceosome components (U1, U2, U4/U6.U5 snRNPs), RNA processing factors (e.g. RNA helicases and m6A regulators), genome integrity maintenance (DNA repair and telomere maintenance proteins), DNA replication components and chromatin remodeling complexes as enriched processes. Overall, this illustrates the general principle that these processes are key regulators of R-loop biology. As further support for the validity of the dataset it is notable that many genes emerging as RLBPs have published roles in R-loop homeostasis, such as the subunits of Mre11-Rad50-Nbs1 (Chang *et al.* 2019), RPA (Nguyen *et al.* 2017), BAF (Bayona-Feliu *et al.* 2021; Tsai *et al.* 2021), and THO complexes (Luna *et al.* 2019). Thus, our machine learning approach effectively recovers known, and predicts novel, R-loop binding and regulating proteins.

### Validation of novel candidate R-loop regulating proteins

While it is important to note that our 2 screens stand alone as they are predicting different proteins based on their training dataset, intersection of their predictions revealed a high-confidence dataset of 288 R-loop modulatory/interacting proteins (Fig. 4c, yellow). Among the genes that overlap between the IP-MS RF and Prox-MS RF datasets are known R-loop regulators involved in RNA processing, such as SF3A3 and SRSF2 (Li and Manley 2005; Tanikawa *et al.* 2016; Singh *et al.* 2020), as well as DNA replication and repair proteins like MCM6 and PARP1 (Vijayraghavan *et al.* 2016; Ye *et al.* 2021). Given the many literature connections to R-loops within this small overlapping group, we consider it a very high confidence set of genes. To validate this classification, we chose 2 additional genes, FXR1 and LIG1, for further study. LIG1 is the major replicative DNA ligase, and a mutant of its yeast ortholog CDC9 was synthetic lethal with RNaseH mutants, and raised S9.6 staining in our previous work (Chang *et al.* 2019). FXR1 on the other hand is an RNA-binding protein and ortholog of the fragile X and mental retardation gene FMR1 (Siomi *et al.* 1995).

**Fig. 6.**
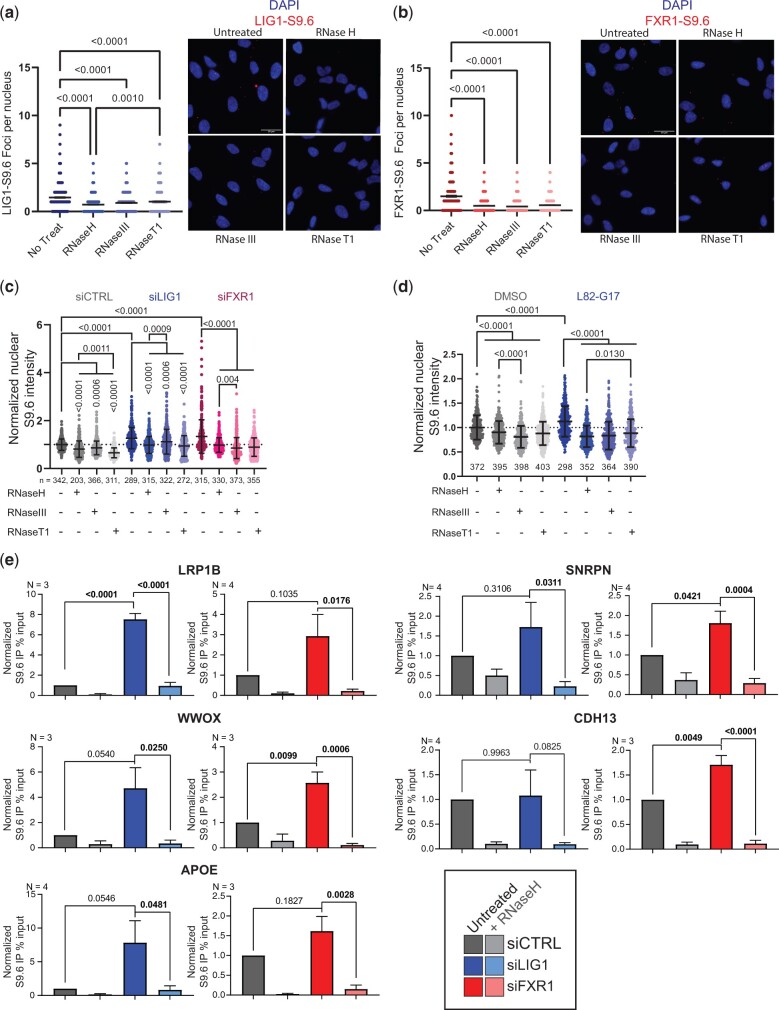
Validation of novel candidate R-loop modulatory proteins. *Left:* quantification of nuclear PLA foci showing localization of LIG1 (a) or FXR1 (b) at sites of S9.6 staining. *Right:* representative images (1-way ANOVA, *N* = 3, >300 nuclei analyzed). Scale bars = 20 µm. See minus primary antibody controls in Supplementary Fig. 3b. c) Quantification of nuclear S9.6 immunofluorescence signal in siRNA knockdown of LIG1 and FXR1. Values are normalized to siCTRL. (mean ± SD, 1-way ANOVA, *N* = 3, number of nuclei analyzed is presented under each distribution). Representative images can be found in Supplementary Fig. 3c. d) Quantification of nuclear S9.6 signal from immunofluorescence showing inhibition of LIG1 with L82-G17 (25 µM, 4 h) results in increased nuclear S9.6 staining (mean ± SD, 1-way ANOVA, *N* = 3, number of nuclei analyzed is presented under each distribution). Representative images can be found in Supplementary Fig. 3d. e) Relative DRIP-qPCR signal values at *LRP1B*, *SNRPN*, *WWOX*, *APOE*, and *CDH13* genes in HeLa cells transfected with indicated siRNAs and treated with in vitro RNaseH preimmunoprecipitation where indicated (mean ± SEM, 1-way ANOVA, at least 3 independent replicates). Statistically significant values shown in bold.

Previous studies on a small scale have identified proteins that can affect R-loop stability using the S9.6 monoclonal antibody (Chan *et al.* 2014; Barroso *et al.* 2019; Chang *et al.* 2019). Other studies have used DNA damage and RNaseH1 expression as a proxy for R-loop accumulation and associated genome instability (Paulsen *et al.* 2009). Antibodies that recognize nucleic acids in a nonsequence specific way are challenging as they can crossreact with other structures. While S9.6 clearly recognizes DNA:RNA hybrid structures in R-loops, these hybrids of course can appear elsewhere (e.g. okazaki fragments, resected DNA ends, retrotransposons, etc.), and S9.6 cross reacts with structured RNAs, especially ribosomal RNA (Smolka *et al.* 2021). Thus, while increases in S9.6 staining can indicate changes in other cellular RNAs more broadly, the staining also can indicate changes in DNA:RNA hybrid levels associated with R-loops. To confirm that these candidate proteins could interact with R-loops, we performed a PLA in HeLa cells to quantify the colocalization of FXR1 or LIG1 at sites of S9.6 staining (Fig. 6, a and b; Supplementary Fig. 3b). In accordance with our data, we were able to observe FXR1-S9.6 and LIG1-S9.6 PLA signals in the form of distinct nuclear foci under native conditions. In both cases, the nuclear signal was reduced when the cells were treated with RNaseH, RNaseIII, and RNaseT1.siRNA depletion of FXR1 or LIG1 in Hela cells led to an increase in nuclear S9.6 staining in both knockdowns compared with the control (Fig. 6c; Supplementary Fig. 3, a and d). Treatments with RNaseH, RNaseIII, and RNaseT1 partially rescued the signal in all datasets, confirming the unspecific nature of the S9.6 antibody. The increase in S9.6 signal in the siLIG1 sample was mostly rescued by RNaseH and RNaseT1, suggesting that knockdown of LIG1 may lead to increased R-loops as well as the accumulation of ssRNA structures within the nucleus. On the other hand, knockdown of FXR1 was partially rescued by RNaseH and significantly reduced by RNaseIII and T1, suggesting that loss of FXR1 may lead to increased levels of R-loops and perhaps to a larger extent dsRNAs and ssRNAs. Additionally, we sought to selectively disrupt LIG1 function using the inhibitor L82-G17, which is an uncompetitive inhibitor of the third step of the ligation reaction phosphodiester bond formation (Howes *et al.* 2017). Inhibition of LIG1 by L82-G17 resulted in an increased nuclear S9.6 staining (Fig. 6d; Supplementary Fig. 3c) that was significantly reduced by RNaseH treatment, suggesting an increase in R-loops. However, RNaseIII and RNaseT1 treatments also reduced the signal intensity, suggesting an increase in the accumulation of dsRNA and ssRNAs species, respectively.

To further confirm that LIG1 and FXR1 knockdown cells accumulate more R-loops and to partly circumvent the limitations associated with the nonspecific nature of immunofluorescence using the S9.6 antibody, we performed DNA:RNA immunoprecipitation followed by qPCR (DRIP-qPCR) at R-loop prone loci *SNRPN*, *APOE*, and *RPL13A* (Ginno *et al.* 2013; Bhatia *et al.* 2014; Herrera-Moyano *et al.* 2014; García-Rubio *et al.* 2015; Barroso *et al.* 2019), as well as the common fragile sites *WWOX*, *LRP1B*, and *CDH13* (Helmrich *et al.* 2011; Okamoto *et al.* 2018). As seen in Fig. 6e, depletion of LIG1 by siRNA resulted in a significant increase at the *LRP1B* locus, and strong increases in signal albeit not statistically significant at the *WWOX* and *APOE* loci. On the other hand, depletion of FXR1 by siRNA led to significant increases in DRIP-qPCR signal at the *WWOX*, *CDH13*, and *SNRPN* loci. No changes were detected at the *RPL13A* locus under either condition (Supplementary Fig. 3e). All of the changes in DRIP signal observed could be rescued by in vitro RNaseH treatment, confirming that the signal was driven by DNA: RNA hybrids. These data suggest that R-loops form at a higher frequency in LIG1 and FXR1 depleted cells, and this occurs at common (e.g. *WWOX*) and distinct genomic sequences (e.g. *APOE*, *LRP1B*, *CDH13*, and *SNRPN*). Ultimately, we conclude that LIG1 and FXR1 likely regulate R-loop accumulation and localize to sites of DNA:RNA hybrids, providing independent validation of our high confidence RLBP predictions.

## Discussion

R-loops have emerged in the past decade as pervasive genome-associated structures with roles in gene expression, RNA processing, telomere maintenance, DNA repair, and epigenetic regulation. The literature suggests that many dozens of proteins likely regulate R-loop formation, function, and dissolution. Here we reasoned that known RLBPs will have conserved features that could be used to implicate new proteins in R-loop biology and help assign each protein in the entire human proteome a score that predicts the ability to associate with R-loops. Our machine learning approaches integrate complementary approaches in IP-MS and Prox-MS to robustly predict RLBPs with functional roles across all of the processes impacted by R-loops. We envision this list as a resource that will interface with the vast and growing number of functional genomic screens taking place in human cell models. For example, screens that capture new genome stability or epigenetic regulators could crossreference the RLBP dataset to determine if R-loop changes are likely mechanisms of action. We can retrospectively state that this approach would be useful. For example, we have previously shown an R-loop mechanism of genome instability when splicing factors like FIP1L1 or EFTUD2 homologues are mutated, both of which appear on our RLBP list (Stirling *et al.* 2012; Tam *et al.* 2019). More recently, our group and others have found that members of the BAF chromatin remodeling complexes play roles in preventing R-loop associated replication stress (Bayona-Feliu *et al.* 2021; Tsai *et al.* 2021) and our RLBP list predicts that both catalytic and accessory BAF subunits are R-loop binding candidates. Additional hits include the DDX23 helicase that plays a crucial role in the regulation of RNAPII pausing to prevent harmful R-loop accumulation (Sridhara *et al.* 2017), PARP1 (also identified in Cristini *et al.*) which functions in the protection against R-loop associated DNA damage through its interactions with DHX9 and TonEBP (Ye *et al.* 2021), and more. In general then, previously recognized R-loop regulators, including those not included in the training set, score very highly in our analysis.

Our machine learning algorithms are built to reduce false positives, and therefore are expected to have an increased false negative rate resulting in potentially missed RLBPs. Features such as the ability to bind nucleic acids and abundance solely rely on annotated databases that are updated frequently. Furthermore, large-scale annotations are not entirely accurate. For example, 2 proteins RnaseH1 and RnaseH2A that bind and resolve R-loops (Cerritelli and Crouch 2009) are not classified as “nucleic-acid binding” within the Uniprot database. Both proteins are classified as “non-R-loop binding” as they score below 0.5 (Supplementary Table 3). However, manually changing their nucleic-acid binding to “true” (or 1) results in a higher score with RnaseH2A crossing the model threshold of 0.5 to be called as an RLBP. Since we use a harsher threshold of 0.8 to enrich for a higher confidence RLBP dataset, RnaseH2A which scores 0.79 gets excluded. However, this also indicates that nucleic-acid binding is not the sole feature for a protein to be called an RLBP and that every other feature used contributes to a protein’s RLBP score (Supplementary Fig. 2, a–i). This indicates that with constant updates, better annotations, and more features being discovered, our approach can be reapplied to increase prediction accuracy.

LIG1 is an ATP-dependent DNA ligase with functions in DNA replication, recombination, and base excision repair, and it was previously shown that LIG1 plays a role in the repair of DNA double strand break lesions associated with R-loop mediated genome instability (Yasuhara *et al.* 2022). Here we show that disruption of LIG1 with RNA interference or with chemical inhibition results in an increase in S9.6 staining and DRIP-qPCR, supporting a functional role in R-loop modulation. However, it is not clear why loss of LIG1 would increase R-loops across the genome. LIG1 plays an essential role in completing synthesis of the lagging strand during DNA replication (Zheng and Shen 2011), and cells depleted for LIG1 recruit 10 times more PARP in S-phase to unligated Okazaki fragments in order to recruit alternative ligases (Hanzlikova *et al.* 2018). Furthermore, LIG1 depletion leads to incomplete Okazaki fragment maturation, which in turn affects ATAD5-dependent unloading of PCNA from chromatin (Kubota *et al.* 2015; Thakar *et al.* 2020). Previous work has shown that PCNA accumulation on chromatin due to ATAD5 loss results in transcription–replication conflicts that lead to R-loop formation behind the replication fork. It is possible that interfering with this LIG1–ATAD5–PCNA pathway could result in increased R-loop formation due to retention of excess PCNA on DNA.

FXR1 is an RNA-binding protein that interacts with the functionally similar protein FMR1, which has a proposed role in physically recruiting the R-loop processing helicase DHX9 to prevent harmful R-loop accumulation and is implicated in R-loop resolution through its interaction with methyl-5-cytosine (m5C) regulators TRDMT1 and TET1 (Chakraborty *et al.* 2021; Yang *et al.* 2022). In addition, both FXR1 and FMR1 are mutated in the R-loop associated disease, Fragile X syndrome (Groh *et al.* 2014; Loomis *et al.* 2014; Chakraborty *et al.* 2020). Here, we show that disruption of FXR1 by RNA interference results in an increase in nuclear S9.6 signal by imaging and DRIP-qPCR which can be partially attributed to R-loops. In addition, we also show that FXR1 colocalizes to sites of S9.6 by PLA, suggesting that FXR1 can be recruited to sites of R-loops. Though the mechanism by which FXR1 impacts R-loop homeostasis remains to be uncovered, we speculate that FXR1 may function in a similar fashion as its ortholog FMR1 as a “detector” of R-loops. Additionally, FXR1 can physically interact with MRE11, which our group has previously identified as important for R-loop suppression by the Fanconi Anemia pathway (Chang *et al.* 2019). This FXR1-MRE11 interaction was shown to be particularly important in the cellular response to ROS (Qi *et al.* 2020), which is known to impact R-loop formation (Teng *et al.* 2018). Future work on this previously unrecognized role of FXR1 in R-loop homeostasis may shed light on the mechanisms underlying the genome instability phenotypes associated with Fragile X syndrome and other R-loop associated trinucleotide expansion diseases.

R-loops functioning as mediators of epigenetic states, in telomere regulation, in DNA repair, and at transcription–replication conflicts mean that they have the potential to be recognized and regulated by many proteins. While dozens of proteins have been ascribed functions in R-loop regulation, the complete spectrum of potential R-loop binding is not understood. This complexity was the ultimate rationale for generation of the R-loop binding resource datasets presented here. Researchers suspecting a relationship between their protein of interest and R-loop biology can use the dataset presented here as a preliminary investigation tool. Moreover, the emergence of S9.6 alternatives that can detect R-loops more specifically will improve researchers’ ability to assess R-loop binding and modulatory functions in the future. For example, the recent use of a purified recombinant catalytically inactive human RNase H1 tagged with GFP shows strong specificity against R-loops (Crossley *et al.* 2021). Refinements to methods and curation of functionally validated RLBPs will help to improve this resource going forward.

## Data availability

Figures 1–6, Supplementary Figs. 1–3 and Supplementary Tables 1–5 can be found on GSA figshare: https://doi.org/10.25387/g3.19782502. Scripts used for RF tuning, development and prediction can be found on Github (https://github.com/arunk95/RLBP_Prediction). Further information and requests for resources and reagents should be directed to and will be fulfilled by the Lead Contact, Peter Stirling (pstirling@bccrc.ca).
